# Identification of a Novel Peptide from *Agaricus blazei* Murrill and Its Immune-Enhancing Activity by Regulation of PI3K/AKT/mTOR Signaling Pathways in RAW 264.7 Cells

**DOI:** 10.3390/foods14203467

**Published:** 2025-10-11

**Authors:** Xianguo Zou, Yeqi Yu, Yun Chi, Kai Yang, Zeyuan Deng, Hong Gu, Peilong Sun

**Affiliations:** 1 College of Food Science and Technology, Zhejiang University of Technology, Hangzhou 310014, China; 2 State Key Laboratory of Food Science and Resources, Nanchang University, Nanchang 330047, China; 3 Genhawk (Wuhan) Biotech Company Limited, 331 Minzudadao, Wuhan 430074, China

**Keywords:** *Agaricus blazei* Murrill, immune-enhancing peptide, molecular mechanism, molecular docking, LNEDELRDA, PI3K/AKT/mTOR

## Abstract

This study aimed to screen and identify a novel immune-enhancing peptide and explore the molecular mechanism. Five novel peptides were identified from *Agaricus blazei* Murrill (ABM), and their secondary structure components consisted of random coil (50.5%), α-helix (28.9%), β-turn (15.6%), and β-sheet (5.0%). A novel peptide (LNEDELRDA) with a molecular weight of 1074.0989 Da could bind with PI3K, AKT, mTOR, IL-6, IL-1β, and TNF-α through hydrogen bonding interactions, and the binding energies were −8.1, −8.3, −7.2, −6.0, −7.4, and −5.8 kcal/mol, respectively. This peptide was synthesized and validated for immune-enhancing ability, showing the strongest immune-enhancing capacity by increasing the cell viability and phagocytic activity of RAW 264.7 macrophages, significantly promoting the production of NO, cytokines TNF-α, IL-1β, and IL-6 in cells, and up-regulating the mRNA and protein expression levels of the PI3K/AKT/mTOR signaling pathway. Our results are the first to reveal that ABM-derived peptide LNEDELRDA could be considered as a promising food-borne immunomodulator that could contribute to enhancing immune function.

## 1. Introduction

The immune system is an important defense system against external pathogens in the human body [1]. In the past few decades, a growing number of individuals have experienced immune dysfunction due to unhealthy lifestyles and infections by viruses or bacteria, predisposing them to the development of multiple forms of disease, such as tumors and inflammation [2]. Currently, chemicals such as pidotimod, thiocarbamate, cyclophosphamide, penicillamine, prostaglandins, and cyclosporine A are commonly used to regulate human immune function [3]. However, high prices and toxic side effects are the main disadvantages of chemical drugs [4]. Hence, against the backdrop of escalating global demand for natural immunomodulatory agents, immune-regulating active peptides derived from natural food sources have gained substantial traction in both academic research and industrial applications. This growing prominence is reflected in remarkable market expansion: the market value of bioactive peptides stood at 48.62 billion USD in 2020 and is projected to hit 95.71 billion USD by 2028, translating to a compound annual growth rate (CAGR) of 8.86% from 2021 to 2028 [5,6,7]. Within this broader market, the application of immunomodulatory peptides is gradually increasing, such as in tumor treatment, vaccine adjuvant, and antibacterial agent [8,9].

*Agaricus blazei* Murrill (ABM), also known as Brazilian mushroom and Komatsu mushroom, is a large-sized fungus commonly grown in China, Brazil (*Cogumelo do Sol*), the United States, and Japan (*Himamatsutake*) [6]. The fruiting body of the macrofungus ABM abounds in protein and amino acids, and the protein content of dried ABM is more than 30%. Its fat and cholesterol content is low, which could help the body to digest and absorb it quickly [10]. Recently, numerous studies have shown that the active ingredients in ABM exhibit lipid-lowering, anti-tumor, and antioxidant properties [11,12,13]. However, domestic studies on active ingredients in ABM mainly focus on the polysaccharides [14]. Limited study has concentrated on the exploitation of peptides from ABM (ABMP) [15]. Feng et al. isolated polypeptides named ABp-RRQ, ABp-RQR, and ABp-TRAR from the fruiting bodies of *Agaricus blazei*. The ABp-RQR (Molecular Weight: 458 Da) serves as the primary bioactive component, exerting anti-inflammatory effects via the modulation of Nrf2 and TLR/NF-κB signaling cascades [15]. However, they did not analyze the sequence of the peptide, and the secondary structure was unclear. In our previous study, we obtained four peptide fractions with different molecular sizes from ABM and confirmed that ABMP-2 with a molecular weight (MW) of 3–5 kDa had the best antioxidant capacity and immunomodulatory ability [16]. However, a key peptide for immunomodulatory activity has not yet been identified. The structure–activity characteristics of immunomodulatory peptides are not fully clear. Collectively, these problems indicate that it is necessary to screen immunomodulatory ABMP and clarify their immunomodulatory mechanism.

Structural identification methods of peptides mainly include mass spectrometry and protein sequence analysis [17]. Molecular docking technology is the latest technology developed in recent years, which can help predict and evaluate the functional activity of peptides. Li et al. used molecular docking to study the interaction between peptides and renin, revealing that peptide segments possessing more charge and hydrophobic character display stronger inhibitory activity toward renin [18]. Molecular docking can also be applied to predict the interaction of peptides with Keap1 through hydrogen bond formation and hydrophobic interaction, providing evidence that a subset of peptides possesses robust cellular antioxidant capacity [19].

At present, studies regarding the immunoregulatory mechanism of peptides mainly concentrate on classical signaling pathways such as MAPK and NF-κB [20,21]. As an indispensable transcription factor, NF-κB is central to the transcriptional control of numerous genes, such as genes encoding pro-inflammatory cytokines TNF-α, IL-1β, and IL-6, and their contents are closely related to the immune function and can exert regulatory effects on the immune response [22,23]. The PI3K/AKT/mTOR is able to change the activation state of downstream immune-related signaling molecules (such as NF-κB) and indirectly participate in cellular immunity [21]. The NVP-BEZ235 is a dual-target PI3K/mTOR inhibitor that could inhibit the activity of PI3K, mTORC1, and mTORC2 by binding to the ATP domain [24]. For this reason, NVP-BEZ235 could serve as a targeted inhibitor to verify whether peptides regulate the immune activity through the PI3K/AKT/mTOR signaling pathway.

Given the underexplored immunomodulatory potential of ABM-derived peptides, this study aimed to purify ABM peptides and characterize their sequence/structure for understanding structure–activity relationships. Molecular docking was then used to precisely screen key immune-enhancing peptides, and the immune-enhancing activity of these peptides was investigated in RAW 264.7 cells. Finally, the immune-enhancing mechanism through PI3K/AKT/mTOR signally pathway was elucidated. Results from this study will provide foundational support for the application of immunoactive peptides from *Agaricus blazei* Murrill.

## 2. Materials and Methods

### 2.1. Materials and Reagents

The dried ABM samples used in this study were furnished by Zhejiang Huihe Biotechnology Co. (Ningbo, China). The mouse macrophage RAW 264.7 cells were provided by the Chinese Academy of Sciences cell bank (Shanghai, China). Penicillin–streptomycin antibodies, phosphate-buffered saline (PBS), fetal bovine serum (FBS), and RPMI-1640 culture medium were obtained from Gibco BRL (Life Technologies Ltd., Paisley, UK). The protease and phosphatase inhibitor, radio immunoprecipitation assay (RIPA) lysis buffer, and PI3K/mTOR dual-target inhibitor NVP-BEZ235 were from Thermo Fisher Scientific (Waltham, MA, USA). Pepsin (3000 U/g protein), 3-(4,5-dimethylthiazol-2-yl)-2,5-diphenyltetrazolium bromide (MTT), dimethyl sulfoxide (DMSO), and lipopolysaccharide (LPS) were purchased from Sigma Chemical Co. (St. Louis, MO, USA). Primary antibodies of β-actin, PI3 Kinase (PI3K), p-PI3 Kinase (p-PI3K), AKT, p-AKT, mTOR, p-mTOR, and secondary antibody anti-rabbit IgG (HRP-linked) were obtained from Cell Signaling Technology (Danvers, MA, USA).

### 2.2. Purification and Isolation of ABMP-2

ABMP-2 was prepared by enzymatic hydrolysis according to our previously published method. In our experiments, four components were isolated from *Agaricus blazei* Murrill, namely ABMP-1 (24.52 ± 0.49 mg), ABMP-2 (84.29 ± 1.17 mg), ABMP-3 (106.94 ±1.049 mg), and ABMP-4 (166.20 ± 5.60 mg). The total mass of ABMP-2 is 101.7 ± 1.02 mg, with a peptide content of 84.29 ± 1.17 mg (purity of 82.88 ± 1.15%). Further purification of ABMP-2 resulted in two components, ABMP-2-P1 and ABMP-2-P2. Five peptides were identified from ABMP-2-P1, and peptide LNEDELRDA, with a yield of 4.00 ± 0.89%, was found to have the best immune activity [16]. ABM powder was prepared by pulverizing the dried samples and subsequently blended with distilled water at a ratio of 1:30 (*w*/*v*). The mixture was first mechanically homogenized and subsequently agitated at 25 °C for 24 h, yielding the supernatant. The supernatant was centrifuged at 10,000 rpm for 15 min (Fresco 17, Thermo Fisher Scientific, Waltham, MA, USA) and precipitated at 4 °C with 85% (NH_4_)_2_SO_4_ overnight. Subsequently, the precipitate was dissolved in distilled water for ultrasonic treatment (360 W, 30 min) by an LCD double frequency ultrasonic cleaning instrument (JM-20D-45/80, Skymen, Shenzhen, China) and then hydrolyzed with 4% pepsin (3000 U/g) at 37 °C. After that, the hydrolysate was passed through pre-washed ultrafiltration membranes and lyophilized to obtain the ABMP-2. Sephadex G-25 was used to purify the ABMP-2 extract. It was dissolved in deionized water to prepare a 2 mg/mL peptide solution, and then was loaded on the Sephadex G-25 column (1.6 × 60 cm), eluting with double-distilled water (ddH_2_O) at a flow rate of 1 mL/min. The absorption peak was ascertained with the Synergy H1 Multi-Mode Microplate Reader (BioTek, Shoreline, WA, USA). Ultimately, the eluent matching the absorption peak at 280 nm was collected and lyophilized.

### 2.3. The FT-IR Analysis of ABMP-2-P1

The determination of FT-IR was performed with reference to the method reported by Beck et al. [25]. 2 mg of dried ABMP-2-P1 and 200 mg of KBr were mixed, ground, and pressed into thin slices with a tablet press, and the slices were scanned over a wavenumber range of 500–4000 cm^−1^ using a 4100 series Fourier transform spectrophotometer (Jasco Inc., Easton, MO, USA).

### 2.4. The CD Analysis of ABMP-2-P1

ABMP-2-P1 at a concentration of 0.5 mg/mL was measured at a wavelength coverage of 190–280 nm, scanning at a speed of 60 nm/min at room temperature (RT) under the protection of nitrogen gas. After processing CD spectral data with the spectra manager software, the proportion of ABMP-2-P1 secondary structure (α-helix, β-sheet, β-turn, and random coil) was analyzed and calculated on DichroWeb (http://dichroweb.cryst.bbk.ac.uk accessed on 5 June 2023) [26].

### 2.5. Mass Spectrometry Analysis of ABMP-2-P1

The peptide sequence was analyzed in accordance with the protocol reported by Zhou [17]; 1200 liquid chromatography electrospray ionization tandem mass spectrometry (LC-ESI-MS/MS) was employed for the identification and analysis of ABMP-2-P1. The chromatographic column was a Zorbax 300SB C18 column (0.3 × 150 mm, 5 μm). A 6538 precision mass spectrometer equipped with an orthogonal electrospray ionization source was used to determine the specific peptides map. The mobile phases were 0.1% formic acid aqueous solution (A) and 0.1% formic acid 84% acetonitrile aqueous solution (B). The gradient elution conditions were 0–50 min, 4–50 % B; 50–54 min, 50–100% B; 54–60 min, holding at 100% B. The elution flow rate was 300 μL/min, and the column temperature was 30 °C. The injection volume was 10 μL. The dry gas temperature was 350 °C, and the gas flow rate was 10 L/min. The atomization gas pressure was 40 psi, and the capillary voltage was 4000 V. The mass spectrum was collected in the range of 50–3000 *m*/*z*. MassHunter workstation B.05.00 software was employed for instrument control and data processing.

### 2.6. Molecular Docking

The previously identified peptides were drawn using ChemDraw 22 software. Docking simulations were performed using AutoDock Vina 1.2.3 (La Jolla, FL, USA). X-ray crystal structures of PI3K (PDB ID: 4ZOP), AKT (PDB ID: 7NH4), mTOR (PDB ID: 4JSV), IL-6 (PDB ID: 1ALU), IL-1β (PDB ID: 3O4O), and TNF-α (PDB ID: 2ZJC) were obtained from RCSB PDB database (http://www.rcsb.org) and then were pretreated by Autodock Vina 1.2.3 software to dehydrate and remove water molecules. The molecular docking of the above receptor proteins to ligand peptides was conducted using AutoDock Vina 1.2.3, and the binding capacity was calculated. The conformation with the minimum docking energy between the receptor and ligand was selected, converted into pdb format using OpenBabel 2.3.1 (OpenEye Scientific, Santa Fe, NM, USA) software, and the 3D optimal configuration and 2D interaction between the receptor peptide and ligand molecule was analyzed using Pymol 2.2.0 (Schrödinger, New York, NY, USA) and Maestro 14.3 (Schrödinger, New York, NY, USA) software.

### 2.7. Peptide Synthesis

A solid-phase peptide synthesis (SPPS) method was employed for the synthesis of peptide LNEDELRDA, conducted by Yuantai Biotech Co., Ltd. (Nanjing, China). The purity of the synthetic peptide was detected by RP-HPLC under following conditions: Mobile phase A, 0.1% trifluoroacetic acid (TFA) in acetonitrile; mobile phase B, 0.1% TFA in water; detection wavelength, 220 nm; flow rate, 1.0 mL/min; column, COSMOSIL Packed Column (4.6 × 250 mm, 5 μm).

### 2.8. Cell Culture and Experimentally Designed Groups

RAW 264.7 cells were maintained in RPMI 1640 medium supplemented with 10% fetal bovine serum (FBS) and 1% penicillin–streptomycin in a humidified atmosphere of 5.0% CO_2_ at 37 °C. The experimental-designed groups were as follows: (1) Control group: cells cultured in routine complete medium containing 10% FBS; (2) LPS group: cells cultured in complete medium containing 10% FBS and LPS (1 μg/mL); (3) Sample groups: cells cultured in complete medium containing 10% FBS and different concentrations of LNEDELRDA (50, 100, 150, 200, 400, 600, 800, 1000 μg/mL); (4) Control + NVP-BEZ235 group: cells cultured in complete medium containing 10% FBS and NVP-BEZ235 (100 nmol/L); (5) LPS + NVP-BEZ235 group: cells cultured in complete medium containing 10% FBS, LPS (1 μg/mL) and NVP-BEZ235 (100 nmol/L); (6) Sample + NVP-BEZ235 group: cells cultured in complete medium containing 10% FBS, different concentrations of LNEDELRDA (50, 100, 150, 200, 400, 600, 800, 1000 μg/mL) and NVP-BEZ235 (100 nmol/L).

### 2.9. Determination of Cell Viability

LNEDELRDA’s cytotoxic effect on RAW 264.7 cells was evaluated by using the classic MTT assay. Cells were inoculated in 96 plates at a density of 1 × 10^4^ cells/mL and cultured according to the above experimental design groups. After incubation, 20 μL of 5 mg/mL MTT reagent was added to each well and incubated for 4 h at 37 °C in the dark. Subsequently, the culture medium was replaced by 150 μL DMSO for each well, and then the formazan was thoroughly dissolved. The absorbance of each well was measured at OD 490 nm by a microplate reader (Biotek Synergy H1, BioTek Instruments Inc., San Diego, CA, USA).

### 2.10. Determination of Cell Phagocytosis

Following the grouping in 2.8, cells in each well were stimulated with LNEDELRDA at concentrations of 50, 100, and 150 μg/mL, respectively. After treatment, 100 µL of 0.075% neutral red solution was added to each well and incubated for 1 h at 37 °C. Then, cells were lysed with a mixture of ethanol and acetic acid (*v*/*v* = 1:1) for 4 h at RT after washing with PBS three times, and OD 540 nm was measured.

### 2.11. Determination of NO and Cytokine Secretion

RAW 264.7 cells (2.5 × 10^5^ cells/mL, 2 mL) were cultured in a 6-well plate and grouped following 2.8, and the cells in each well were stimulated with LNEDELRDA at concentrations of 50, 100, and 150 μg/mL, respectively. The supernatant was collected, and the levels of NO were measured using Griess reagent (Beyotime Biotechnology, Shanghai, China), and the levels of IL-6, IL-1β, and TNF-α were detected using ELISA kits (Jingmei Biotechnology Co., Shenzhen, China) according to the manufacturers’ instructions. All cytokine measurements were performed with 3 independent biological replicates using RAW 264.7 cells from a separate passage to account for inter-sample variability. For each biological replicate, supernatant samples were analyzed in 3 technical replicates to minimize intra-assay error. All assays were conducted under the same standard operating procedure (SOP).

### 2.12. Western Blot Analysis

After cleaning the RAW 264.7 cells thrice with pre-cold PBS in the 6-well plates, cells were lysed for 10 min with 1 mL/well RIPA buffer (with 1% phosphatase and 1% protease inhibitor). The lysates were collected and centrifuged at 10,000 r/min for 15 min using a pre-cooled centrifuge at 4 °C, and the supernatant was collected for subsequent experiments. The protein concentrations of supernatants were determined by a BCA protein assay kit (Beyotime Biotechnology, Shanghai, China). Then, the protein of each sample was mixed with 5× loading buffer and boiled for 10 min. Protein lysates (20 μg) were loaded into and separated by SDS-PAGE (6% SDS-PAGE for mTOR and p-mTOR, 10% SDS-PAGE for other proteins), then transferred onto PVDF membranes. After incubating in blocking buffer (5% nonfat milk in TBST) for 1 h, the membranes were then incubated with primary antibodies of β-actin, PI3K, AKT, mTOR, p-PI3K, p-AKT, and p-mTOR overnight at 4 °C, and then incubated with secondary antibodies for 1 h after cleaning membranes with TBST three times. Finally, protein bands were visualized using an ECL developer (Beyotime Biotechnology, Shanghai, China) and imaged with an automatic digital gel imaging analysis system (Tanon, Shanghai, China).

### 2.13. Quantitative Real-Time PCR

Total RNA was collected using FastPure Cell Total RNA Isolation Kit V2 (Vazyme, Nanjing, China), and the cDNA was prepared according to the manufacturer’s kit method of HiScript II Q RT SuperMix (Vazyme, Nanjing, China). The process was carried out using a method of SYBR Green I fluorescence inlay, referring to the q-PCR kit (Vazyme, Nanjing, China), and the 2^−ΔΔCT^ method of calculation was used for mRNA expression. The forward and reverse primers of the PI3K, AKT, mTOR, IL-6, IL-1β, and TNF-α genes were listed in Table 1.

### 2.14. Statistical Analysis

All experiments were performed in independent triplicates to mitigate experimental variability, and data are expressed as mean ± standard deviation (SD). Intergroup differences in mean values were assessed via one-way analysis of variance (ANOVA) followed by Tukey’s honestly significant difference (HSD) post hoc test, utilizing SPSS 17.0 statistical software. Statistical significance was denoted as *p* < 0.05 (*), *p* < 0.01 (**), and *p* < 0.001 (***).

## 3. Results and Discussion

### 3.1. Purification of ABMP-2

Our previous study showed that four ABMPs were obtained by ultrafiltration of ABM, all of which have good antioxidant capacity and immunomodulatory ability, among which ABMP-2 (Molecular Weight: 3–5 kDa) has the best effect [16]. In this study, the immunomodulatory mechanism of ABMP-2 was elucidated, and its primary and secondary structures were analyzed.

**Figure 1:**
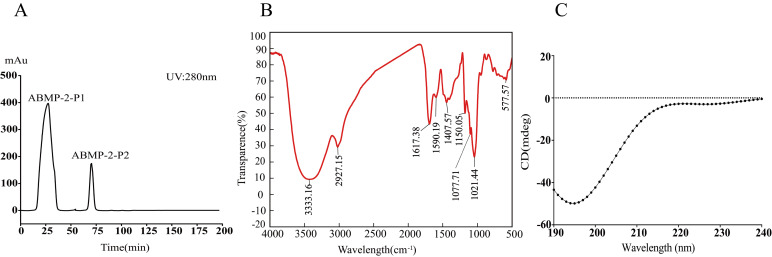
Separation of ABMP-2 (**A**) and structural identification (**B**,**C**) of ABMP-2-P1 from ABMP-2. (**A**) Separation by Sephadex G-25 column; (**B**) FT-IR spectrum; (**C**) CD spectra.

Gel filtration chromatography is widely used to separate peptides based on molecular weight, having the advantages of high reproducibility, high recovery, simple operation, and mild separation conditions [27]. As shown in Figure 1A, ABMP-2 was separated using Sephadex G-25, and two peptide components were obtained, named ABMP-2-P1 and ABMP-2-P2. ABMP-2-P1, with the highest peak area, was first eluted, followed by ABMP-P2 with lower content. The results suggested that the main peptide component in ABMP-2 was ABMP-2-P1; hence, ABMP-2-P1 was chosen for subsequent structural analysis and immunomodulatory investigation.

### 3.2. Characterization of ABMP-2-P1

#### 3.2.1. Secondary/Tertiary Structure of ABMP-2-P1 by FT-IR Spectroscopy

The bonds of proteins and peptides are amide groups, and studies have shown that the peptide structure can be determined by the amides I, II, III, and A bands in the FT-IR absorption peaks. Among them, the peak characterizations of amides I, II, III, and A bands in infrared spectra are 1600–1700 cm^−1^, 1500–1600 cm^−1^, 1400–1100 cm^−1^, and 3300–3500 cm^−1^, respectively [28]. As shown in Figure 1B, the infrared spectrum of ABMP-2-P1 in the range of 500–4000 cm^−1^ was scanned to analyze the functional groups. The infrared spectrum of ABMP-2-P1 shows a primary amide absorption peak at 3333.16 cm^−1^ caused by N-H stretching vibration. There is a peak at 1617.38 cm^−1^ attributed to the stretching vibration of C = O in the amide I band, resulting from the presence of p-π conjugation between electron pairs on the nitrogen atom and carbonyl group. In addition, the infrared spectrum at 1590.19 cm^−1^ represented the amide II band, resulting from the coupling between δNH and V_C-H_, and at around 1150.05 cm^−1^ showed the amide III band [29,30]. All these results indicated that ABMP-2-P1 exhibits the characteristic peaks of peptides and has a relatively complete peptide structure.

#### 3.2.2. Secondary Structure of ABMP-2-P1 by CD Spectroscopy

Circular Dichroism spectroscopy is widely employed to characterize the secondary structural conformations of macromolecules (e.g., proteins and peptides), encompassing α-helix, β-sheet, β-turn, and random coil [31]. As shown in Figure 1C, the largest negative peak appears around 200 nm, indicating that ABMP-2-P1 exhibits an irregular curly structure [32]. Meanwhile, indistinct negative peaks around 208 nm and 222 nm were found, suggesting that ABMP-2-P1 may contain a small amount of α-helix structure [33]. As listed in Table 2, ABMP-P1 was identified with random coil, α-helix, β-turn corners, and β-sheet, accounting for 50.5%, 28.9%, 15.6%, and 5.0%, respectively. Therefore, the main structure of ABMP-2-P1 is random coil, α-helixl, and β-turn corners.

### 3.3. Primary Sequence of ABMP-2-P1 Using LC-MS/MS

**Figure 2:**
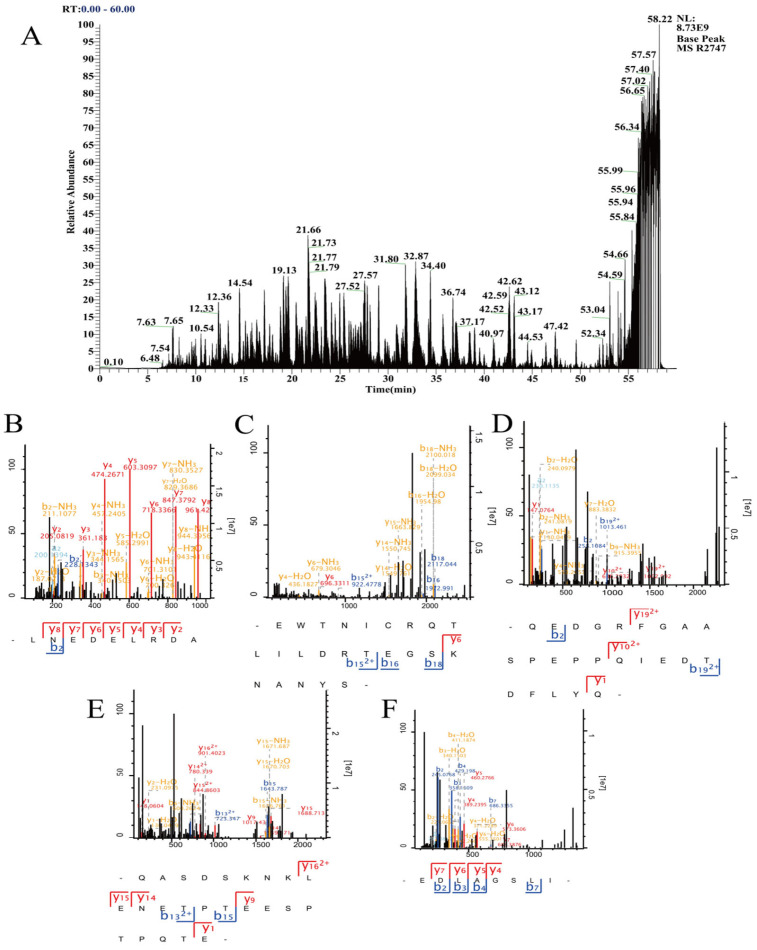
Basepeak diagram of ABMP-2-P1 (**A**) and MS/MS spectrum (**B**–**F**) of identified top five novel peptides from ABMP-2-P1. (**B**) LNEDELRDA; (**C**) EWTNICRQTLILDRTEGSKNANYS; (**D**) QEDGRFGAASPEPPQIEDTDFLYQ; (**E**) QASDSKNKLENETPTEESPTPQTE.

Mass spectroscopy is a commonly used peptidomics analysis tool with the characteristics of high throughput and high resolution [17]. In this study, the molecular mass and amino acid sequences of ABMP-2-P1 were analyzed and identified by LC-MS/MS. As shown in Figure 2, ABMP-2-P1 is composed of 46 peptides, and the sequences, molecular weights, and percentages of the top five novel peptides were retrieved and are listed in Table 3, namely LNEDELRDA (1074.099 Da, 12.2%), EWTNICRQTLILDRTEGSKNANYS (2811.361 Da, 10.7%), QEDGRFGAASPEPPQIEDTDFLYQ (2709.219 Da, 7.0%), QASDSKNKLENETPTEESPTPQTE (2659.209 Da, 6.4%), and EDLAGSLI (816.423 Da, 5.9%). The amino acid residue numbers of these five peptides ranged from 8 to 24, with MW between 800 and 2900 Da. It has been reported that low-molecular-weight peptides (MW less than 2000 Da) have significant immunomodulatory activity. Li obtained peptides with MW between 459.3 and 2716.3 Da from natural marine products, and these peptides were found to have the effect of inhibiting pathogenic bacteria in the intestines and increasing beneficial bacteria in the intestines [34]. He et al. demonstrated that low-molecular-weight (<1 kDa) peptides derived from the thick shell mussel (*Mytilus coruscus*) could markedly promote the proliferation and phagocytic activity of RAW 264.7 cells, thereby facilitating cytokine release [35]. Therefore, peptide LNEDELRDA with a MW of 1074.099 and the highest content as well as highest credibility scores was selected for a subsequent exploration of immune regulatory activity.

### 3.4. Interaction Between LNEDELRDA and Immune-Related Protein Targets Using Molecular Docking

Molecular docking techniques are capable of predicting docking sites and possible intermolecular interaction bonds [36]. Therefore, docking analysis was conducted to analyze the interaction between LNEDELRDA and active sites of immune-related protein targets (PI3K, AKT, mTOR, IL-6, IL-1β, and TNF-α), respectively. Table 4 presents the interaction energy, hydrogen bond count, amino acid binding sites, and immune-associated targets.

**Figure 3:**
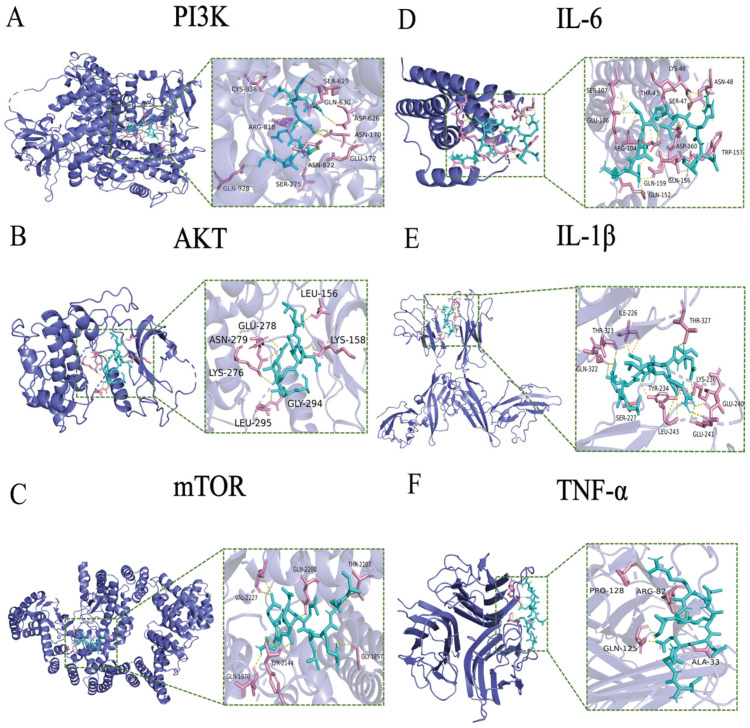
Optimal docking results between LNEDELRDA and immune-related targets. (**A**) PI3K; (**B**) AKT; (**C**) mTOR; (**D**) IL-6; (**E**) IL-1β; (**F**) TNF-α.

The optimal binding configuration for the docking of LNEDELRDA with immune-related proteins is shown in Figure 3. LNEDELRDA is highly associated with PI3K, AKT, mTOR, IL-6, IL-1β, and TNF-α, and the calculated minimum binding energies were −8.1, −8.3, −7.2, −6, −7.4, and −5.8 kcal/mol, respectively, suggesting that LNEDELRDA could significantly act on immunomodulatory proteins. Among them, TNF-α has the highest binding energy (−5.8 kcal/mol), indicating that the binding ability of LNEDELRDA to TNF-α active sites is weaker.

The key binding sites of LNEDELRDA to the PI3K protein were mainly Asn, Gln, and Ser, to the AKT protein, they were mainly Leu and Lys, to the mTOR protein, mainly Gln, to the IL-6 protein, mainly Gln, to the IL-1β protein, mainly Leu and Thr, and to the TNF-α protein, Pro, Arg, Gln, and Ala.

**Figure 4:**
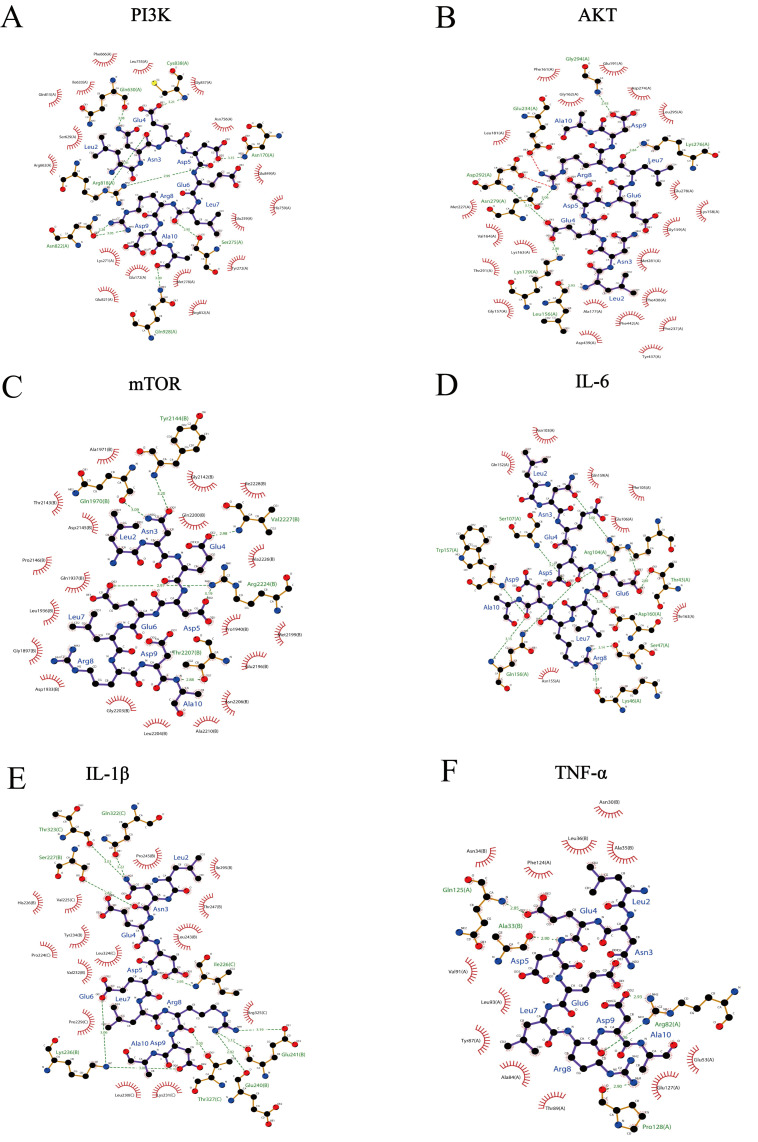
The hydrogen bonds formed between LNEDELRDA and immune-related targets. (**A**) PI3K; (**B**) AKT; (**C**) mTOR; (**D**) IL-6; (**E**) IL-1β; (**F**) TNF-α.

The hydrogen bonds formed between LNEDELRDA and immune-related targets are shown in Figure 4. Specifically, those between LNEDELRDA and PI3K were at amino acid residues of Asn170, Ser275, Gln630, Arg818, Asn822, Cys838 and Gln928; those between LNEDELRDA and AKT were at amino acid residues of Leu156, Lys179, Glu234, Lys276, Asn279, Asp292 and Gly294; those between LNEDELRDA and mTOR were at amino acid residues of Gln1970, Tyr2144, Thr2207, Arg2224 and Val2227; those between LNEDELRDA and IL-6 were at amino acid residues of Thr43, Lys46, Ser47, Arg104, Ser107, Gln156 and Trp157; those between LNEDELRDA and IL-1β were at amino acid residues of Ile226, Ser227, Lys236, Glu240, Glu241, Gln322, Thr323 and Thr327; and those between LNEDELRDA and TNF-α were at amino acid residues of Ala33, Arg82, Gln125 and Pro128. The above molecular docking results indicated that LNEDELRDA exhibited a strong binding affinity to key immune-related proteins involved in the PI3K/AKT/mTOR signaling pathway. It is speculated that LNEDELRDA might activate the PI3K/AKT/mTOR signaling pathway to exert immune-enhancing activity. Numerous studies have indicated that most of the amino acids with immunomodulatory effects are hydrophobic, with the most common ones being valine (Val), glycine (Gly), leucine (Leu), phenylalanine (Phe), proline (Pro), glutamic acid (Glu), tyrosine (Tyr), negatively charged amino acids, and aromatic amino acids. In addition, the peptides containing Ile and Ala are able to promote the proliferation of immune cells and stimulate the production of immune-associated factors [37]. The content of the above-mentioned amino acids in peptides LNEDELRDA, EWTNICRQTLLILDLTEGSKNANYS, QEDGRFGAASPEPPQIEDTDFLYQ, QASDSKNKLENETPTEESPTQTE, and EDLAGSLI is 77.78%, 45.83%, 75%, 41.67%, and 87.5%, respectively. Peptide LNEDELRDA contains high levels of hydrophobic amino acids. Therefore, we investigated whether LNEDELRDA exhibited an immune-enhancing effect through PI3K/AKT/mTOR using the RAW 264.7 cells model.

### 3.5. Synthesis and Identification of LNEDELRDA

**Figure 5:**
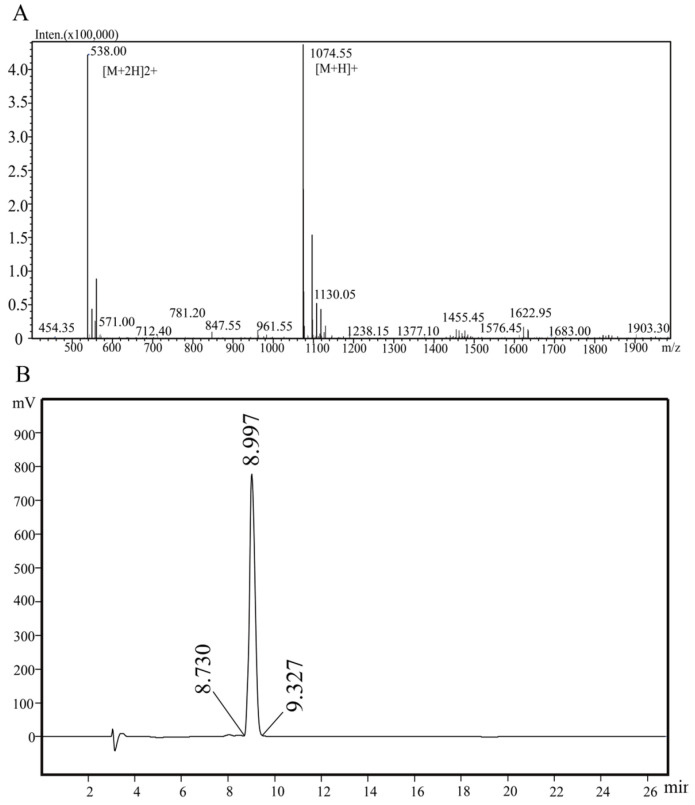
Chromatographic analysis of LNEDELRDA. (**A**) LC-MS/MS; (**B**) HPLC.

As shown in Figure 5A,B, according to the amino acid sequence, LNEDELRDA was synthesized, and its molecular weight and sequence were identified by HPLC and mass spectrometry, showing a molecular weight of 1074.10 Da and purity of 97.4%.

### 3.6. Effect of LNEDELRDA on Cell Viability of RAW 264.7 Macrophage

**Figure 6:**
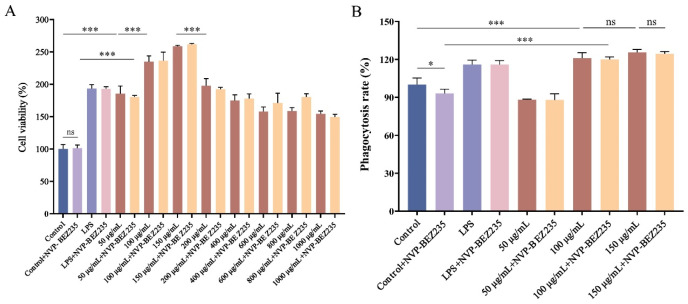
Effect of different concentrations of LNEDELRDA added with or without inhibitor NVP-BEZ235 on RAW 264.7 macrophage. (**A**) cell viability; (**B**) cell phagocytosis. Significances between two groups (ns *p* > 0.05; * *p* < 0.05; *** *p* < 0.001) were listed in the Figure.

The results of LNEDELRDA on the viability of RAW 264.7 cells are shown in Figure 6A. Within a concentration range of 50–150 μg/mL, the cell viability significantly increased with increasing concentration (*p* < 0.001), reaching a maximum value of 258.6 ± 1.54%, even higher than that of the positive LPS group (193.4 ± 6.01%). When the concentration of LNEDELRDA exceeded 150 μg/mL (200–1000 μg/mL), the cell viability significantly decreased to a value of 50 μg/mL. As a dual-target inhibitor, NVP-BEZ235 towards PI3K/mTOR was used for studying immune-enhancing effects and related mechanisms in the following experiments. The viabilities of NVP-BEZ235 and NVP-BEZ235 + LNEDELRDA on RAW 264.7 cells were also determined. As shown in Figure 6A, cell viability between the control + NVP-BEZ235 group and the control group (*p* > 0.05) showed no significant difference. In addition, cell viabilities of NVP-BEZ235 added with different concentrations of LNEDELRDA at 50–150 μg/mL showed a significant upward trend (*p* < 0.001), and the value was similar to that of the LNEDELRDA group without NVP-BEZ235, indicating that the NVP-BEZ235 inhibitor and LNEDELRDA had no cytotoxic effect on the survival rate of the RAW 264.7 macrophage. Hence, LNEDELRDA within a concentration range of 50–150 μg/mL can promote RAW 264.7 macrophage activation, and was selected for subsequent experimental studies.

### 3.7. Effect of LNEDELRDA on Cell Phagocytosis of RAW 264.7 Macrophage

Macrophages serve as the host’s primary line of defense against invading pathogens and exert a pivotal role in the innate immune system. During the immune response, macrophages emerge as one of the primary cell types to initiate a response [38,39]. The effect of LNEDELRDA on the phagocytic ability of RAW 264.7 cells was investigated using a neutral red phagocytosis assay, and the results are shown in Figure 6B. At a concentration of 50 μg/mL, LNEDELRDA did not strengthen the phagocytic ability of RAW 264.7 macrophages. When the concentration of LNEDELRDA reached 100 and 150 μg/mL, the cell phagocytic rate significantly increased (*p* < 0.001), reaching 120.9 ± 4.39% and 125.5 ± 2.39%, respectively, which was even more effective than that of the positive LPS group (116.0 ± 3.52%). The results showed that LNEDELRDA at a concentration of 100–150 μg/mL can enhance the phagocytic activity of RAW 264.7 macrophages.

Interestingly, with the addition of inhibitor NVP-BEZ235 (targeting PI3K/mTOR), the phagocytosis rate of the control + NVP-BEZ235 group was significantly lower than that of the control group (*p* < 0.05), indicating that the inhibitor NVP-BEZ235 suppressed the phagocytosis capacity of macrophages. In addition, the cell phagocytosis rates of the LNEDELRDA + NVP-BEZ235 group at 100 and 150 μg/mL were significantly (*p* < 0.001) higher than those of the control + NVP-BEZ235 group and the control group, and the effect was equivalent to that of LNEDELRDA at the same concentrations, indicating that LNEDELRDA can still promote the phagocytic capacity, even in NVP-BEZ235 inhibitor-treated RAW 264.7 cells. This indicates that LNEDELRDA exhibiting immune-enhancing activity might be through PI3K/AKT and other signaling pathways, such as Toll-like receptor (TLR) signaling and mitogen-activated protein kinase (MAPK) pathways. For example, Wang et al. found that polysaccharides from *Collybia radicata* mushrooms could activate macrophages, enhance the phagocytosis ability of cells, and induce the release of inflammatory factors via the TLR4 signaling pathway [40]. In addition, Shen et al. indicated that polysaccharides from *Hibiscus sabdariffa* Linn. are able to activate macrophages through MAPKs and NF-κB signaling pathway [41]. Therefore, we speculate that LNEDELRDA might exert immune-enhancing activity through multiple signaling pathways.

### 3.8. Effect of LNEDELRDA on NO Secretion in RAW 264.7 Macrophage

**Figure 7:**
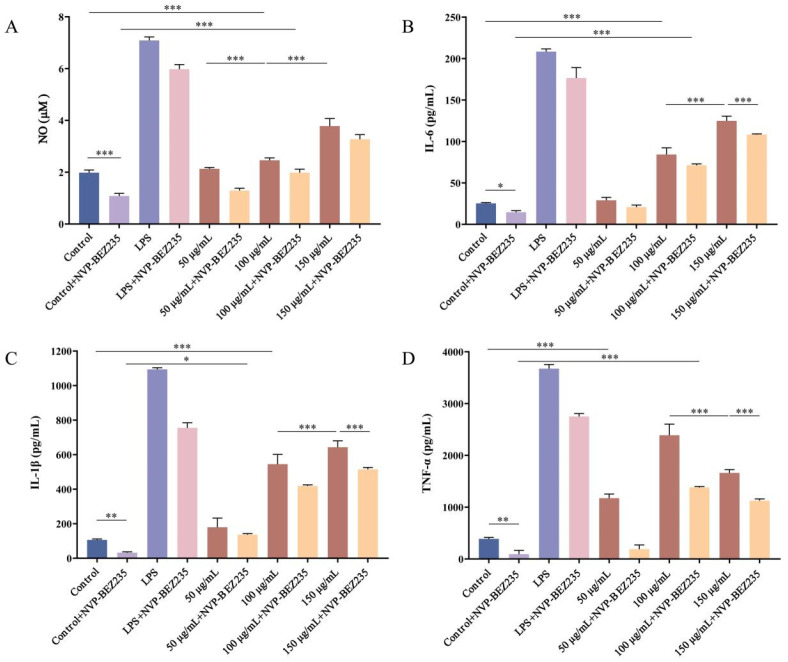
Effect of LNEDELRDA added with or without inhibitor NVP-BEZ235 on NO and cytokine secretions of RAW 264.7 cells. (**A**) NO; (**B**) IL-6; (**C**) IL-1β; (**D**) TNF-α. Significances between two groups (* *p* < 0.05; ** *p* < 0.01; *** *p* < 0.001) were listed in the Figure.

The production of NO is one of the important characteristics of macrophage activation by stimulation, which is closely related to the inflammatory response of cells [42]. The impact of LNEDELRDA on NO secretion in RAW 264.7 cells is depicted in Figure 7A. The experimental results showed that as the concentration of LNEDELRDA increased from 50 to 150 μg/mL, the secretion of NO in cells increased, reaching its maximum value (3.78 ± 0.29 μM) at 150 μg/mL. After the addition of PI3K/mTOR inhibitor NVP-BEZ235, the NO secretion of the control + NVP-BEZ235 group was significantly lower than that of the control group (*p* < 0.001), suggesting that the inhibitor NVP-BEZ235 can inhibit macrophages from producing NO. With the treatment of LNEDELRDA, the cellular NO secretion level in the LNEDELRDA + NVP-BEZ235 group at 100 and 150 μg/mL was significantly higher than that of the control + NVP-BEZ235 group and the control group (*p* < 0.001), reaching a maximum of 3.27 ± 0.18 μM. However, the cellular NO level in all LNEDELRDA groups with NVP-BEZ235 inhibitor added was markedly lower than that of the uninhibited group (*p* < 0.001). The above results indicate that LNEDELRDA could promote NO secretion even in NVP-BEZ235 inhibitor-treated RAW 264.7 cells. Meanwhile, it has been hypothesized that NVP-BEZ235 inhibited NO production through suppressing the activation of the PI3K/AKT/mTOR signaling pathway. Lee investigated the anti-inflammatory effects of a tryptophan metabolite (3-HAA) on macrophages and explored its molecular mechanism and found that 3-HAA significantly inhibited NO production in LPS-stimulated RAW 264.7 cells and also down-regulated the protein expressions of the PI3K/AKT/mTOR signaling pathway [42]. This is consistent with our experimental results.

### 3.9. Effect of LNEDELRDA on Cytokine Secretion in RAW 264.7 Macrophage

In addition to producing NO, activated macrophages also release a range of inflammatory cytokines (such as IL-6, IL-1β, and TNF-α) that contribute to pathogen killing and phagocytosis. IL-1β plays a significant regulatory role in the occurrence of inflammatory and autoimmune diseases [43]. IL-6 has various functions, such as promoting B cell maturation, T cell activation, and lymphocyte typing [44,45]. TNF-α is one of the key pro-inflammatory cytokines secreted by macrophages, exerting functions in immune regulation and tumor cell inhibition [46]. The effects of LNEDELRDA on the secretion of the above cytokines in RAW 264.7 cells were shown in Figure 7B–D. Compared with the control group, the addition of LNEDELRDA significantly increased the levels of secretory factors IL-6, IL-1β, and TNF-α. With the concentration of LNEDELRDA increasing from 50 to 150 μg/mL, the secretion levels of IL-6 and IL-1β gradually increased, reaching the maximum values at 150 μg/mL, 124.86 ± 5.67 pg/mL, and 642.81 ± 37.32 pg/mL, respectively. In particular, TNF-α reached its maximum value (2388.75 ± 214.33 pg/mL) at an LNEDELRDA concentration of 100 μg/mL.

After the addition of the dual-target inhibitor NVP-BEZ235, the secretion of IL-6, IL-1β, and TNF-α in the control + NVP-BEZ235 group was significantly lower than that in the control group (*p* < 0.05), indicating that NVP-BEZ235 had a certain inhibitory effect on the secretion of the above cytokines, especially IL-1β and TNF-α. With the treatment of LNEDELRDA, the secretions of IL-6 and IL-1β in LNEDELRDA + NVP-BEZ235 groups at 50–150 μg/mL significantly increased, reaching a maximum value of 108.31 ± 0.81 pg/mL and 514.69 ± 10.82 pg/mL at 150 μg/mL, respectively. However, the maximum secretion of TNF-α was found at an LNEDELRDA concentration of 100 μg/mL, which may be due to the fewer binding sites between LNEDELRDA and the TNF-α protein. When the concentration of LNEDELRDA continued increasing, the binding sites were saturated, which might have resulted in a decrease in TNF-α secretion, which was consistent with the results of molecular docking. It is worth noting that the secretion levels of cytokines in all LNEDELRDA groups with NVP-BEZ235 inhibitor added were significantly lower than those without the addition of NVP-BEZ235 inhibitor (*p* < 0.001), which was in accordance with the results of the NO secretion.

The above experimental results show that PI3K/mTOR inhibitor NVP-BEZ235 could inhibit the production of cytokines IL-6, IL-1β, and TNF-α in RAW 264.7 macrophages. LNEDELRDA within a concentration range of 50–150 μg/mL could effectively promote the secretion of the above cytokines, even in NVP-BEZ235 inhibitor-treated macrophages. In addition, the above results of LNEDELRDA and/or NVP-BEZ235 on the secretions of NO and cytokines suggest that LNEDELRDA might exert immune-enhancing activity through PI3K/mTOR signaling pathways. The ability of NVP-BEZ235 to suppress basal secretion of IL-6, IL-1β, and TNF-α aligns with the well-established roles of the PI3K/mTOR pathway in regulating macrophage cytokine production. Previous studies have demonstrated that PI3K/mTOR signaling controls inflammatory cytokine synthesis through modulation of transcription factors such as NF-κB, as well as translational regulation of cytokine mRNAs. Specifically, mTOR inhibition has been shown to reduce IL-6 and TNF-α secretion in macrophages, consistent with our findings in RAW 264.7 cells [47,48,49].

### 3.10. Effect of LNEDELRDA on Modulating the mRNA Expression Levels of PI3K/mTOR in RAW 264.7 Cells

**Figure 8:**
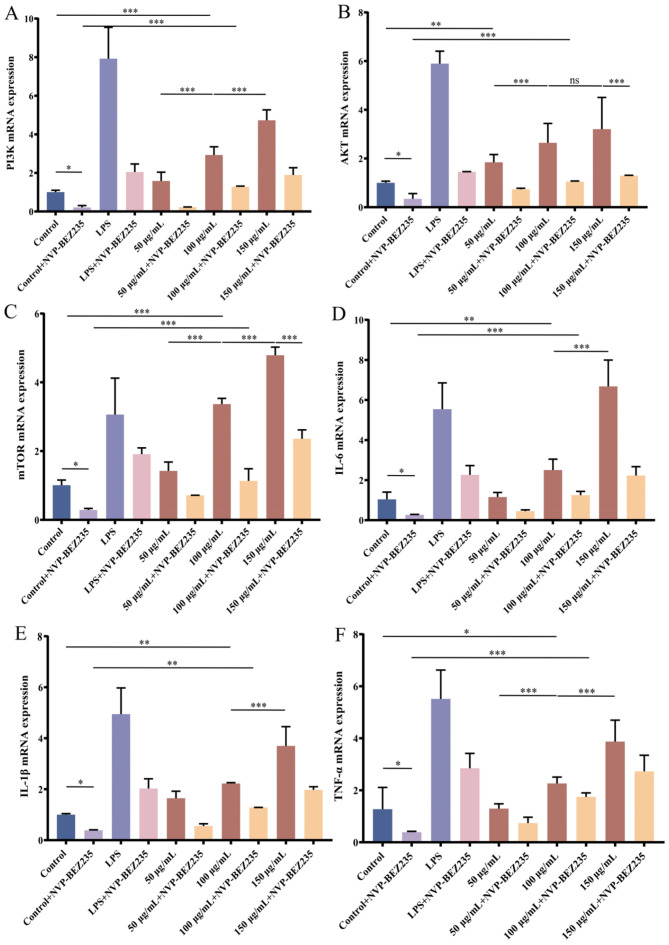
Effects of LNEDELRDA added with or without inhibitor NVP-BEZ235 on mRNA expression of RAW 264.7 cells. (**A**) PI3K; (**B**) AKT; (**C**) mTOR; (**D**) IL-6; (**E**) IL-1β; (**F**) TNF-α. Significances between two groups (* *p* < 0.05, ** *p* < 0.01, *** *p* < 0.001) were listed in the Figure.

In order to further verify the immune response effect of LNEDELRDA on RAW 264.7 macrophages, the mRNA expression levels of the PI3K/mTOR signaling pathway (PI3K, AKT, mTOR) as well as cytokine-regulating genes (IL-6, IL-1β, TNF-α) were observed in RAW 264.7 cells. As shown in Figure 8, the mRNA expression levels of PI3K, AKT, mTOR, IL-6, IL-1β, and TNF-α were significantly upregulated when the concentration of LNEDELRDA was 100 to 150 μg/mL compared with the control group (*p* < 0.001), reaching their maximum value at a concentration of 150 μg/mL. Dual-target inhibitor NVP-BEZ235 towards PI3K/mTOR significantly inhibited the mRNA expression of PI3K/mTOR signaling pathway and cytokine-regulating genes in the control, positive LPS, and all LNEDELRDA experimental groups, leading to speculation that LNEDELRDA played an immune-enhancing role via the modulation of the PI3K/AKT/mTOR signaling pathway.

### 3.11. Effect of LNEDELRDA on Regulating the Protein Expressions of the PI3K/AKT/mTOR Pathway

**Figure 9:**
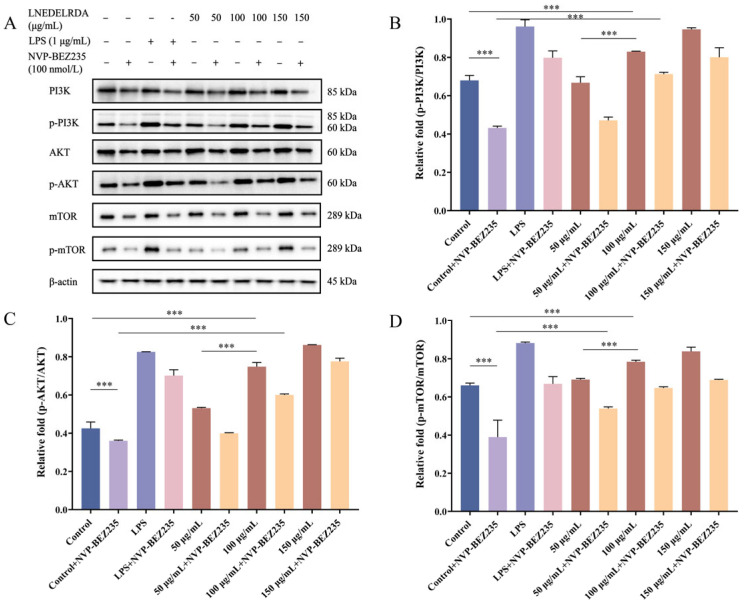
Effects of LNEDELRDA added with or without inhibitor NVP-BEZ235 on PI3K/AKT/mTOR signaling pathway. (**A**) Protein expression band of PI3K/AKT/mTOR signaling pathway and its phosphorylated protein; (**B**) the ratio of p-PI3K/PI3K protein; (**C**) the ratio of p-AKT/AKT protein; (**D**) the ratio of p-mTOR/mTOR protein. Significances between two groups (*** *p* < 0.001) were listed in the Figure.

In order to further verify that LNEDELRDA exerted immune-enhancing activity through the PI3K/AKT/mTOR pathway, the protein expressions of PI3K, AKT, mTOR, and their phosphorylated levels were detected by Western blot, and the results are shown in Figure 9. LNEDELRDA at concentrations of 100 and 150 μg/mL was able to significantly induce the up-regulation of phosphorylated PI3K, AKT, and mTOR proteins, and the effect of LNEDELRDA at 150 μg/mL was even higher than that of the positive control LPS group. The ratios of p-PI3K/PI3K, p-AKT/AKT, and p-mTOR/mTOR in RAW 264.7 cells incubated with LNEDELRDA were significantly increased in a dose-dependent manner. However, the addition of inhibitor NVP-BEZ235 significantly decreased the phosphorylated protein levels of PI3K, AKT, and mTOR in the control, positive LPS, and all LNEDELRDA experimental groups, demonstrating that LNEDELRDA indeed exerted immune-enhancing effects through regulating the PI3K/AKT/mTOR signaling pathway.

Taken together, LNEDELRDA at concentrations of 100 and 150 μg/mL exhibited potent immune-enhancing activity in RAW 264.7 macrophages through activating the PI3K/AKT/mTOR signaling pathway, which was evidenced by their strong binding ability, improved cell survival rate and phagocytosis rate, promotion of NO and cytokine (IL-6, IL-1β, and TNF-α) secretion, increase of the mRNA expression levels of PI3K, AKT, mTOR, IL-6, IL-1β, and TNF-α, and up-regulation of p-PI3K, p-AKT, and p-mTOR protein expression levels.

**Figure 10:**
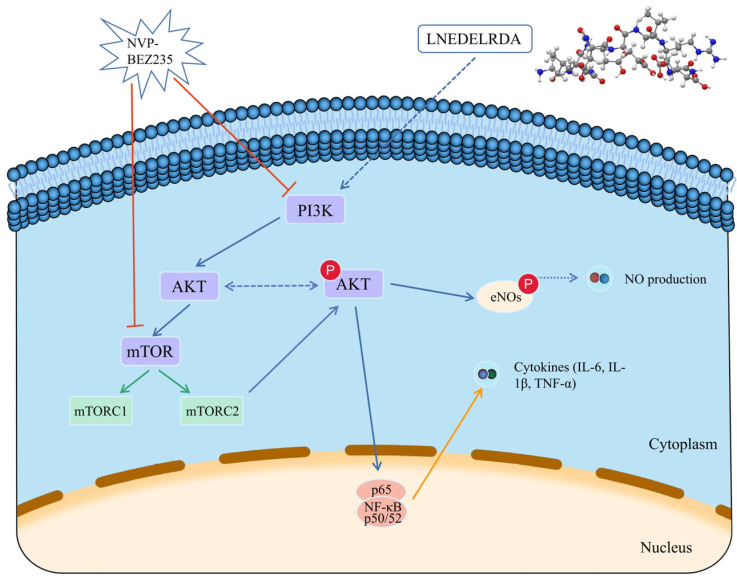
The possible immune-enhancing mechanism of LNEDELRDA through the PI3K/AKT/mTOR signaling pathway.

The PI3K/AKT/mTOR signaling pathway is widely involved in cell proliferation, differentiation, apoptosis, autophagy, and metabolism [50]. PI3K plays a critical role in intracellular signaling; it will be converted into the second messenger phosphatidylinositol phosphate (PIP3) when activated by growth factors, cytokines, etc [51,52]. AKT is a downstream signaling molecule of PI3K and plays a central role in the PI3K/AKT/mTOR signaling pathway. It could be fully activated by the generated PIP3. Activated AKT is transferred from the cell membrane to the cytoplasm or nucleus, then continues to target downstream signaling molecules for regulation, such as mTOR and NF-κB [53]. mTOR belongs to the kinase family related to phosphatidylinositol kinase, converging multiple signaling pathways [54]. Inhibition of mTOR is capable of suppressing NF-κB activation and inflammatory factor release (IL-6, IL-1β, TNF-α). Studies have shown that certain medicines can inhibit the activation of NF-κB by suppressing the PI3K/AKT/mTOR signaling pathway [53,54,55]. Accordingly, we put forward the proposition that the PI3K/AKT/mTOR signaling pathway may activate the NF-κB pathway to facilitate the secretion of inflammatory cytokines. Wang speculated that Ginsenoside Rg1 regulates macrophage innate immune response by differentially regulating NF-κB and PI3K/AKT/mTOR pathways in RAW 264.7 cells and mouse peritoneal macrophage experiments [55]. Li found that *Pinellia ternata* lectin is able to regulate the NF-κB signaling pathway by mediating inflammation and activating PI3K/AKT/mTOR [54]. Yu et al. and Wang et al. isolated an active peptide KSPLY from *Hericium erinaceus* by ultrafiltration combined with multistage chromatography separation and identification technology, showing immunomodulatory effects through the TLR4/NF-κB signaling pathway, and facilitating the transformation of M0 macrophages into M1 macrophages as well as M2 macrophages into M1 macrophages at a concentration of 100 μmol/L. In addition, although LNEDELRDA found in our study and KSPLY isolated from *Hericium erinaceus* have immunomodulatory effects, they enhance immunity through different signaling pathways [1,56]. The possible mechanism is concluded and outlined in Figure 10.

## 4. Conclusions

In summary, a novel peptide, LNEDELRDA, with immune-enhancing activity derived from ABM, was screened by combining LC-MS/MS, molecular docking, and in vitro cell experiments. Molecular docking and RAW 264.7 cell experiments proved that LNEDELRDA at concentrations of 100–150 μg/mL exhibited effective immune-enhancing capacity through strong hydrogen binding with PI3K, AKT, mTOR, IL-6, IL-1β, and TNF-α, increasing the cell viability and phagocytic activity, and the activation of the PI3K/AKT/mTOR signaling pathway as well as secretions of NO and cytokines TNF-α, IL-1β, and IL-6 in cells. This study demonstrated that the ABM-derived peptide LNEDELRDA exhibits in vitro immune-enhancing activity, thereby offering preliminary insights and a theoretical basis for the high-value exploitation of ABM and the development of food-sourced immunostimulants. In the future, we will use animal studies to confirm the immunomodulatory activity and safety of LNEDELRDA before translating this immunomodulatory peptide from *Agaricus blazei* Murrill into nutraceutical applications.

## Figures and Tables

**Table 1:** Primer sequences for quantitative RT-PCR.

Genes	Forward Sequences (5′ to 3′)	Reverse Sequences (5′ to 3′)
PI3K	ACACCACGGTTTGGACTATGG	GGCTACAGTAGTGGGCTTGG
AKT	ATGAACGACGTAGCCATTGTG	TTGTAGCCAATAAAGGTGCCAT
mTOR	ACCGGCACACATTTGAAGAAG	CTCGTTGAGGATCAGCAAGG
IL-6	CCAAGAGGTGAGTGCTTCCC	CTGTTGTTCAGACTCTCTCCCT
IL-1β	GCAACTGTTCCTGAACTCAACT	ATCTTTTGGGGTCCGTCAACT
TNF-α	GACGTGGAACTGGCAGAAGAG	TTGGTGGTTTGTGAGTGTGAG
β-actin	AGGGAAATCGTGCGTGACAT	AAAAGAGCCTCAGGGCATCG

**Table 2:** Secondary structure of ABMP-P1 and its proportions.

Peptide Structure	Relative Content (%)
α-helix	28.9
β-sheet	5.0
β-turn	15.6
Random coil	50.5
Total	100.0

**Table 3:** The top five peptide sequences of ABMP-2-P1 were identified by LC-ESI-MS/MS.

Number	Amino Acid Sequences	Peptide Length	Molecular Weight (Da)	Proportion (%)	Credibility Scores
1	LNEDELRDA	9	1074.099	12.2	122.18
2	EWTNICRQTLILDRTEGSKNANYS	24	2811.361	10.7	13.07
3	QEDGRFGAASPEPPQIEDTDFLYQ	24	2709.219	7.0	13.69
4	QASDSKNKLENETPTEESPTPQTE	24	2659.209	6.4	13.35
5	EDLAGSLI	8	816.423	5.9	118.01

**Table 4:** Molecular docking results of LNEDELRDA with immune-related targets.

Target Protein	Binding Energy (kcal/mol)	Number of Hydrogen Bonds	Binding Sites
PI3K	−8.1	9	Asn170, Ser275, Gln630, Arg818, Asn822, Cys838, Gln928
AKT	−8.3	6	Leu156, Lys179, Glu234, Lys276, Asn279, Asp292, Gly294
mTOR	−7.2	6	Gln1970, Tyr2144, Thr2207, Arg2224, Val2227
IL-6	−6	9	Thr43, Lys46, Ser47, Arg104, Ser107, Gln156, Trp157
IL-1β	−7.4	10	Ile226, Ser227, Lys236, Glu240, Glu241, Gln322, Thr323, Thr327
TNF-α	−5.8	5	Ala33, Arg82, Gln125, Pro128

## Data Availability

The original contributions presented in this study are included in the article. Further inquiries can be directed to the corresponding authors.
